# Supplementary material for: Knowledge and attitudes toward complete diagnostic autopsy and minimally invasive autopsy: A cross-sectional survey in Hanoi, Vietnam
Source: PLOS Glob Public Health. 2023 Mar 17;3(3):e0001685. doi: 10.1371/journal.pgph.0001685 (PMC10022770; doi:10.1371/journal.pgph.0001685)
Supplement: Attachment — Submitted filename: PLOS GPH 38HN survey Response Reviewers.docx [file pgph.0001685.s002.docx]

Dear Dr Sadia Shakoor,

Thank you very much for sending the comments for our manuscript. We have edited the manuscript according to the editors’ and reviewers’ comments.

Thank you again.

**Journal Requirements:**

1. Please amend your detailed Financial Disclosure statement. This is published with the article. It must therefore be completed in full sentences and contain the exact wording you wish to be published.

a. Please clarify all sources of funding (financial or material support) for your study. List the grants (with grant number) or organizations (with url) that supported your study, including funding received from your institution. 
b. State the initials, alongside each funding source, of each author to receive each grant.

Added in the submission system.

2. Please provide separate figure files in .tif or .eps format.

Thank you, we have added in the appropriate formats.

3. We have noticed that you have uploaded Supporting Information files, but you have not included a list of legends. Please add a full list of legends for your Supporting Information files after the references list.

Added.

Please review your reference list to ensure that it is complete and correct.

Done.

**Responses to reviewers**

We really appreciate your comments and suggestions. They were really helpful to improve the quality of our paper. Our responses to your comments are as below.

Reviewer #1: 

Abstract
reads in some sentences a little clumsy – please carefully improve language if possible

Some sentences in the Abstract have been revised as indicated. The content of the Abstract was also updated to fit the additional statistical analysis.

M&M
- The sentence “Since we aimed to obtain initial understandings of what people know and think of CDA and MIA, instead of examining the awareness and attitudes of the general public, no formal sample size calculation was done.” is not clear to me.

The sentence is revised to “*The goal of the questionnaire was to obtain initial understanding of what people know and think about CDA and MIA, instead of examining a representative sample that reflects the awareness and attitudes of the whole population, no formal sample size calculation was done.”*

Results
- Socio-demographical characteristics: it would be really interesting to know – in comparison – how representative this is regarding the population in Vietnam in terms of age groups, religion and education. Maybe something could be added in this direction? This would help to be able to follow in the discussion later on why these results are only of limited use.

We have added a paragraph discussing about how representative the sample was in comparison to the whole population as below.

*“First and foremost, it is worth highlighting that due to the sampling methods, the study sample might not be representative, therefore, the results presented here might not be able to reflect the general perceptions of the whole Vietnamese population. The percentages of the participants who were younger than 40 years old and achieved at least university education were higher than national estimates. More than 60% of the participants were young, under 40, while only around 30% of the population were adults under 40 years old (15). Further, the majority of the respondents had at least a university degree, whereas in the general population only 58.1% of Vietnamese people finished high school (16). More than half of the participants included in this study lived in the two biggest cities of the country while according to the national statistics, in 2021, only around 18% of the population lived in these cities (15). The proportion of participants who did not practice any religion was relatively similar to the national statistics of 73.6% (17).”*

- In general, the authors should consider to analyse all their findings comparatively for the different groups. It might be good to split the data into HCW and non-HCW, as it is later, in the discussion, established that HCW often have reservations regarding CDA/MIA because they don’t feel comfortable performing it or even are stigmatized for performing it. This makes these two groups difficult to compare. For the other group that the authors call community the differences among the age groups, education level, site of residents would be highly interesting. Even if data is not enough for statistical testing, it could simply be descriptive (tables).

Since the qualitative study following this survey aims at the HCW and the community groups specifically, we have run additional analyses for all findings for these two groups. However, to make sure the paper does not look exhaustive of tables and figures, some comparative findings were just described in words instead. Below are the tables and figures that compare between the HCWs and the community, with reference to the statistics of the whole sample.

Socio-demographic characteristics of the study sample

| **Socio-demographic variables** | | **Healthcare workers**  **(n_1_ = 74)** | **Community (n_2_ = 320)** | **Total**  **(N = 394)** |
| --- | --- | --- | --- | --- |
|  | | Mean (SD) | Mean (SD) | Mean (SD) |
| **Age** | | 33.6 (8.7) | 37.9 (12.7) | 37.09 (12.2) |
|  | | n (%) | n (%) | n (%) |
| **Age groups** | Under 30 | 28 (40.0) | 92 (28.7) | 120 (30.5) |
|  | 30-39 | 31 (41.8) | 98 (30.7) | 129 (32.7) |
|  | 40-49 | 12 (16.2) | 80 (25.0) | 92 (23.3) |
|  | 50 and above | 3 (4.0) | 50 (15.6) | 53 (13.5) |
| **City/Province of residence** | Hanoi/HCMC | 39 (52.7) | 166 (51.9) | 205 (52.0) |
|  | Other cities/provinces | 35 (47.3) | 154 (48.1) | 189 (48.0) |
| **Education** | Secondary school | 0 (0.0) | 40 (12.5) | 40 (10.2) |
|  | High school | 0 (0.0) | 99 (30.9) | 99 (25.1) |
|  | Undergraduate/  Postgraduate | 74 (100.0) | 181 (66.6) | 255 (64.7) |
| **Religion** | None | 67 (90.5) | 234 (73.1) | 301 (76.4) |
|  | Buddhism | 5 (6.8) | 71 (22.2) | 76 (19.3) |
|  | Christianity | 2 (2.7) | 15 (4.7) | 17 (4.3) |

Benefits of CDA, as perceived by the participants

%

|  | HCWs | Community | All |
| --- | --- | --- | --- |
| Hereditary disease detection | 48.6 | 52.2 | 51.5 |
| Infectious disease detection | 52.7 | 45 | 46.4 |
| Occupational disease detection* | 56.8 | 38.4 | 41.9 |
| CoD determination* | 87.8 | 68.4 | 72.1 |
| Contribution to science | 41.9 | 30.6 | 32.7 |
| No benefit perceived | 0 | 1.9 | 1.5 |

Situations when CDA should be performed

%

|  | HCWs | Community | All |
| --- | --- | --- | --- |
| Suspected deaths | 81.1 | 65.6 | 68.5 |
| Death without known causes | 87.8 | 67.5 | 71.3 |
| When requested by the authority | 75.7 | 43.4 | 49.5 |
| When requested by the family | 68.9 | 47.5 | 51.5 |

For the community group, we chose to perform additional descriptive analyses in some parts to clarify the possibly different perceptions across the socio-demographic groups as indicated in the following responses. The proportions were calculated against the total number of participants in the community group (N=320) to enable comparisons across different domains in the table.

- Reasons for opposing against CDA: It would be interesting to know the distribution of the reasons amongst the different groups.

We have performed additional descriptive analysis on this and included this in the manuscript as shown in the table below.

|  | | **Religion/ Traditions/ Customs violation** | **Body invasion** | **Delay to burial plans** | **Trust in initial clinical diagnoses** | **Knowing CoD is unnecessary** |
| --- | --- | --- | --- | --- | --- | --- |
|  |  | n (%) | n (%) | n (%) | n (%) | n (%) |
| **Age group** | Under 30 | 55 (17.2) | 67 (20.9) | 24 (7.5) | 6 (1.9) | 26 (8.1) |
|  | 30-39 | 54 (16.9) | 70 (21.9) | 19 (5.9) | 11 (3.4) | 18 (5.6) |
|  | 40-49 | 31 (9.7) | 62 (19.4) | 10 (3.1) | 7 (2.2) | 11 (3.4) |
|  | 50 and above | 18 (5.6) | 37 (11.6) | 4 (1.2) | 6 (1.9) | 7 (2.2) |
| **Residence** | Hanoi/HCMC | 93 (29.1) | 135 (42.2) | 38 (11.9) | 17 (5.3) | 39 (12.2) |
|  | Other cities/ provinces | 65 (20.3) | 101 (31.6) | 19 (5.9) | 13 (4.1) | 23 (7.2) |
| **Education** | Secondary school | 9 (2.8) | 32 (10.0) | 3 (0.9) | 4 (1.2) | 3 (0.9) |
|  | High school | 46 (14.4) | 67 (20.9) | 14 (4.4) | 9 (2.8) | 15 (4.7) |
|  | Undergraduate/ Postgraduate | 103 (32.2) | 137 (42.8) | 40 (12.5) | 17 (5.3) | 44 (13.8) |
| **Religion** | None | 121 (37.8) | 165 (51.6) | 47 (14.7) | 18 (5.6) | 50 (15.6) |
|  | Buddhism | 31 (9.7) | 58 (18.1) | 8 (2.5) | 11 (3.4) | 11 (3.4) |
|  | Christianity | 6 (1.9) | 13 (4.1) | 2 (0.6) | 1 (0.3) | 1 (0.3) |

- Preference regarding consent to either CDA or to MIA: This seems to be one of the most crucial questions! Should be elaborated more on, also on the differences observed in the different strata of the surveyed sample population (e.g. age, residence, HCW).

We have added descriptive analysis on consent to either CDA or MIA for the socio-demographic subgroups within the community group. The results are as shown in the table below.

|  | | **Consent to CDA** | **Consent to MIA** |
| --- | --- | --- | --- |
|  |  | n (%) | n (%) |
| **Age group** | Under 30 | 77 (24.1) | 73 (22.8) |
|  | 30-39 | 77 (26.6) | 69 (24.1) |
|  | 40-49 | 63 (19.7) | 68 (21.2) |
|  | 50 and above | 37 (11.6) | 45 (14.1) |
| **Residence** | Hanoi/HCMC | 136 (42.5) | 139 (43.4) |
|  | Other cities/ provinces | 126 (39.4) | 124 (38.8) |
| **Education** | Secondary school | 30 (9.4) | 32 (10.0) |
|  | High school | 83 (25.9) | 81 (25.3) |
|  | Undergraduate/ Postgraduate | 149 (46.6) | 150 (46.9) |
| **Religion** | None | 190 (59.4) | 188 (58.8) |
|  | Buddhism | 58 (18.1) | 64 (20.0) |
|  | Christianity | 14 (4.4) | 11 (3.4) |

Discussion
- It would be better to first discuss the (non-)representativeness of the surveyed sample population for the general public, before comparing the results with other studies and arguing about observed similarities (because the found distributions might be very biased).

We have elaborated this in our Discussion as shown in the response above.

Reviewer #2: Dear Sir/Mad.,
REVIEWER COMMENTS ON: “KNOWLEDGE AND ATTITUDES TOWARD COMPLETE DIAGNOSTIC AUTOPSY AND MINIMALLY INVASIVE AUTOPSY: A CROSS-SECTIONAL SURVEY IN HANOI, VIETNAM”.
The research topic is a good one and the authors’ zeal to help find solution to an identified gap in medical diagnosis is highly recommendable. It is a study that would add to knowledge acquisition. However, the authors have made many typographical errors that would affect the beauty of this important study. Some recommendations are given in continues prose form under each heading, which when modified would help to improve the quality of the paper for publication.

Abstract
In the background of the abstract, insert “the” between the “Knowing” and “cause” of …
Under “Methods/Results” the following editing should be done: Insert “used” in between “study” and “was” so that it becomes: The “study used was cross-sectional” … The authors did not a single questionnaire. So for the same sentence first sentence, they should remove the “a”, make the “questionnaire” plural and also change “was” to “were” and also replace “in” after hospital with a comma “,”. That sentence could now become: “The study used was cross-sectional, using semi-structured questionnaires that were disseminated via several websites and as paper-based in a national level hospital, Vietnam.”
The second sentence should be re-phrased. Then, “were” should be removed from the third (3rd) sentence: “We included 394 surveys were in the analysis.” The words “an”, “issue” and “for” in the last sentence under the conclusion part of the abstract should be edited by removing “an”, changing issue” to “issues” and “for” to “against”.

We have revised most of the errors that were pointed out. We did revise some places differently. We did not change the sentence “The study was cross-sectional” since it is read okay to us.

Introduction
The phrase “Knowledge of cause of death” in the first (1st) sentence under the introduction should be modified by adding “the” between the “of” and “cause”. For the first (1st) sentence of paragraph two (2), a full stop should be placed after “specimens”. After that the first letter that follows it should start another sentence by writing it in capital “For”. Place a comma “,” after “encephalitis” and “particular” then a semi colon after the second specimen before “whereas”.
The second sentence: “The reasons for this are threefold: most deaths occur outside the health system (patients are often discharged to die at home when prognosis is unfavorable), there are no facilities or trained personnel for full autopsy and subsequent pathological and microbiological examination in most provincial and referral hospitals, and a final major factor limiting consent to autopsy are negative attitudes toward post-morten examination, due to religious beliefs and cultural traditions.” in the fourth (4th) paragraph is too long. It should be re-phrased.
The fourth (4th) word “this” and the sixth (6th) word “threefold” should be changed into plural with a full stop placed after “unfavorable” in the bracket. “Secondly”, should be placed between the first word that follows “unfavorable”: “there”, with a full stop “.” replacing “comma” after “hospitals”. Then, “lastly” or “finally” should replace “and” with a comma, then the remaining words that end at “traditions”. References should be provided after it. Starting from “Minimally invasive autopsy …”, move them down to start a new paragraph. Full stop should be after “CDA”. Then “recent” should be started as “recently” with a comma “,” and a full stop “.” after the reference indicate “(6)”. The word “and” should be removed. Then, the first letter of the next word “the” should start with as capital. It should start as: “The Pneumonia Etiology Research …”
Starting from “Both studies …” to “open autopsy”, there should be a full stop after “autopsy” with the first letter of “using” started as capital. The first sentence of paragraph five (5) which would be paragraph six after the fourth paragraph is divided into two (2); should be divided into two sentences. In the seventh (7th) paragraph (or eighth, 8th) if paragraph is divided, the in-text reference: “Le Van Loi and Hoang Thi Lan (2019)” should be sited using the format for in-text referencing. Rephrase sentences that are too long especially those that ere highlight as yellow.
Autopsy is known to be associated with so many negative perceptions. Though demystifying people’s perception about autopsy is not one of the authors’ specific objectives, it would be advisable to tell readers what autopsy is.

We have rewritten some sentences to make it clearer for readers. We have also edited some errors shown. We have also added references to some sentences that were lacking. Definition of complete diagnostic autopsy was added: “*Complete diagnostic autopsy (CDA) is considered the gold standard to establish CoD. The method involves observations of both the outside and the inside of the body, alongside open dissection to acquire tissues from the organs (6). Several relevant analyses would then be performed on the collected samples, including toxicology, microbiology, genetics, etc (6).* ”

Materials and Methods
Use the same recommendation given in the abstract for the questionnaires under this headline.

Edited.

Results and Discussions
A Table 1 of “Soci-demographical characteristics of the participants” is given. The authors have only listed or indicated the various demographical characteristics but they did not use any of them as example to let readers understand why the table is necessary. Authors should briefly explain in at least two or three sentences how the demographic characteristics of the respondents influence their knowledge and attitude about CMA and MIA. They should use some of the demographic attributes of the respondents as examples in their explanations.

We have added more descriptions on why these socio-demographic characteristics were chosen and what they would be used for later in the paper in the Methods section. *“Following a thorough literature review, characteristics such as age, residential location, educational level and religion might affect an individual’s perception on death, dying and the body. These socio-demographic factors, therefore, were included to enable comparisons between groups later on.”*

The authors also stated that “394 surveys were included in the analysis” but this figure cannot be identified from table 1. To solve this issue a row each should added to each group/variable to take care total number of participants in each group. Per their explanation, it means that “394” respondents were used for the survey. So, the total of each group or variable like “Age, City/Province, Education, Occupation” or “Religion” should be “394”. The total for “City/Province, Education”, and “Religion” were each “394” which is correct. But for the two variables, the total for each exceeded “394”. If there is/are any explanation(s) for these, they should be provided.
The next issue with table 1 too is that, they have tried to categorize the respondents, which is a good idea. But their first category “18-30” is not of the same intervals like the rests. Authors should re-categorize their respondents to ensure that they have equal intervals or they can use “below 30” to avoid the issue of unequal intervals.
Just like in Table 1, a row each should be added each variable/group for Tables 2-6 too. Then, the total percentages for each variable/group must be hundred (%). If there is/are any reason(s) why the percentages for variable must exceed (%), then they must be made known to the readers. In the same way, the total for each variable is supposed to be “394”. If there is/are any reason(s) why the total must exceed this number; then readers must know about that.
The same issues raised about the tables are applicable to the figures (Fig. 1, and Fig. 2.). Authors should ensure that the total of each variable, like respondents for the various categories under “Age” or “Religion” is “394”. If the total exceeds “394”, clear explanation(s) should be given to the readers.

The issues were probably because we chose to present the results with one decimal place. We have re-run the analyses and checked each column to make sure the totals are consistent. For each table, we also added the total number on which the percentage was calculated to avoid confusion.

The authors have done well with discussions that provided them with clues for suggestions or recommendations. However, there are still some modifications that need to be done to make their more standardized and understanding. The second sentence of paragraph one (1), under the discussion should be re-phrased to help make it more meaningful. For the first paragraph, authors should compare their findings to what literature says and cite the source of literature. The first two words in the third (3rd) sentence of paragraph two (2) should be re-phrased. Example, it could be “Some participants” or “For some participants”/respondents from our study …

We dedicated the first paragraph of the Discussion to summarize the findings. To avoid confusion, we have added some phrases to clarify this. In the Discussion, we have revised some sentences to make them clearer to readers. “*In summary, most respondents perceived the benefits of CDA to determine CoD and detect hereditary diseases for early treatment….*”

Conclusion, Recommendations or Suggestions
Though the authors have done well by giving a very good conclusion, one of the major reasons why research is done is the ability to give suggestions or recommendations based on the findings to help solve a particular problem. Their results and discussions have provided many clues that they can be used for suggestions or recommendations. Example, if “The MIA method was not well known among respondents”, what is the likely suggestion or recommendation? Or, if amongst MIA and CDA; your respondents are saying that the “MIA might more acceptable”, which of the two would be recommended for stakeholders?
The authors add a sub-heading: “Suggestions/Recommendations” after they have concluded. Then, they should provide suggestions or recommendations to help stake holders to decide what they can do to solve the problem.

We have added a section for Recommendations separately and after the Discussion. Since there will be a qualitative study conducted after this survey which aims to further explore the socio-cultural aspects surrounding consent to CDA and MIA, we did not provide detailed recommendations on this.

***“RECOMMENDATIONS***

*Future studies on similar topics should explore further the socio-cultural barriers against CDA and MIA across different groups with representative samples that would enable comparisons between groups and between the sample and the whole population. The concerns of participants described in this study are also helpful for the development of public engagement materials to increase the awareness and acceptability of MIA among the general public.”*
